# Advances and challenges in understanding evolution through genome comparison: meeting report of the European Molecular Biology Organization (EMBO) lecture course “Evolutionary and Comparative Genomics”

**DOI:** 10.1093/bioadv/vbaf223

**Published:** 2025-10-08

**Authors:** Athina Gavriilidou, Alexandros Stamatakis, Anne Kupczok, Iliana Bista, Chris D Jiggins, Rosa Fernández, Eirini Skourtanioti, Grigoris Amoutzias, Daniela Delneri, Nikos Kyrpides, Christoforos Nikolaou, Alexandros A Pittis, Tereza Manousaki, Nikolaos Vakirlis

**Affiliations:** Department of Computational Biology, University of Lausanne, Lausanne CH-1015, Switzerland; Swiss Institute of Bioinformatics, Amphipôle, Quartier UNIL-Sorge, Lausanne CH-1015, Switzerland; Biodiversity Computing Group, Institute of Computer Science, Foundation for Research and Technology—Hellas, Heraklion, Crete GR-70013, Greece; Computational Molecular Evolution Group, Heidelberg Institute for Theoretical Studies, Heidelberg 69118, Germany; Institute for Theoretical Informatics, Karlsruhe Institute of Technology, Karlsruhe 76131, Germany; Bioinformatics Group, Wageningen University, Wageningen 6708 PB, The Netherlands; Senckenberg Research Institute and Natural History Museum, Frankfurt 60325, Germany; Wellcome Sanger Institute, Tree of Life, Wellcome Genome Campus, Cambridge CB10 1SA, United Kingdom; Department of Zoology, University of Cambridge, Cambridge CB2 3EJ, United Kingdom; Metazoa Phylogenomics Lab, Institute for Evolutionary Biology (CSIC-UPF), Barcelona 08003, Spain; Department of Archaeogenetics, Max Planck Institute for Evolutionary Anthropology, Leipzig 04103, Germany; Bioinformatics Laboratory, Department of Biochemistry and Biotechnology, School of Health Sciences, University of Thessaly, Larissa GR-41334, Greece; Manchester Institute of Biotechnology, Faculty of Biology Medicine and Health, The University of Manchester, Manchester M1 7DN, United Kingdom; DOE Joint Genome Institute, Lawrence Berkeley National Laboratory, Berkeley, CA 94720, United States; Institute for Bioinnovation, Biomedical Sciences Research Centre “Alexander Fleming”, Vari 16672, Greece; Institute of Molecular Biology and Biotechnology of the Foundation for Research and Technology Hellas (IMBB-FORTH), Heraklion GR-70013, Greece; Institute of Marine Biology Biotechnology and Aquaculture, Hellenic Centre for Marine Research, Crete, Heraklion 70014, Greece; Hellenic Pasteur Institute, Athens GR-11521, Greece

## Abstract

This perspective outlines emerging trends, key challenges, and future opportunities in evolutionary and comparative genomics. Our starting point are the topics presented at the 2024 EMBO Early Career Lecture Course “Evolutionary and Comparative Genomics”, which highlighted recent conceptual and methodological advances in areas ranging from microbial pangenomes, protein evolution, hybrid speciation, novel gene origination and transposon dynamics. Here, we emphasize the role of computational and molecular approaches, providing a forward-looking view on where the field is headed and how it is being reshaped by new technologies and approaches.

## 1 Introduction

In the past quarter century, the increasing availability of genomes from organisms across the tree of life has revolutionized evolutionary biology. This revolution shows no signs of fading as technological advancements provide scientists with new “omes,” such as genomes, interactomes, metabolomes, translatomes, or single-cell transcriptomes. These can readily be leveraged to investigate evolutionary processes more accurately and more deeply, opening up entirely new possibilities. As we peel back one layer of genome complexity to only uncover another, established comparative and evolutionary methodologies applied to different elements of the genome yield new insights. At the same time, an avalanche of innovative algorithms, clever optimizations, and theoretical concepts lead to further progress, as exemplified in the deep learning boom that is in the process of transforming research across the life sciences. In this context, opportunities for students and early career researchers working in genomics, evolution and all adjacent fields, to come together with leading experts are valuable and necessary. With this rationale in mind, the first ever EMBO Early Career Lecture Course on “Evolutionary and Comparative Genomics” was held in November 2024, in Nafplion, Greece (link to event website).

This event had a two-fold motivation: first, to provide early career researchers (mostly PhD candidates and postdoctoral researchers) from around the world with an accessible path to absorbing cutting-edge science and present their work in the vibrant discipline of evolutionary and comparative genomics. Additionally, this meeting aspired to generate some much-needed momentum for the Greek evolutionary and genomics research community by providing local early-career researchers with an affordable opportunity to interact with leading experts.

Overall, 18 nationalities were represented, with participants coming from academic institutions in Europe, the Americas, and Asia. Diversity also characterized the scientific content: as hoped for, the course covered a wide range of evolutionary and comparative genomics themes in terms of organism diversity, scope, and methodologies. Invited lectures ranged from population-level approaches to those focusing on entire genera, phyla, or even kingdoms, and from vertebrates, insects or worms, to fungi, viruses, and bacteria.

Thanks to generous support from EMBO, on the day following the main event in Nafplion, we held a 1-day satellite symposium at the Biomedical Sciences Research Center “Alexander Fleming,” in Vari (near Athens, Greece). Open to the local research community and free to attend, this symposium complemented the main event by exploring how genomic processes shape adaptation and complexity across diverse organisms. While some talks revisited themes from the main event—offering valuable insights to a new audience—the symposium primarily provided a platform for fresh perspectives, spanning fungi, viruses, plants, humans, and other animals. It thus allowed participants who did not fit or could not attend the main event to benefit from lectures that were repeated as in the main event, entirely new lectures by invited speakers, as well as additional lectures by stellar early career researchers.

**Figure 1.**
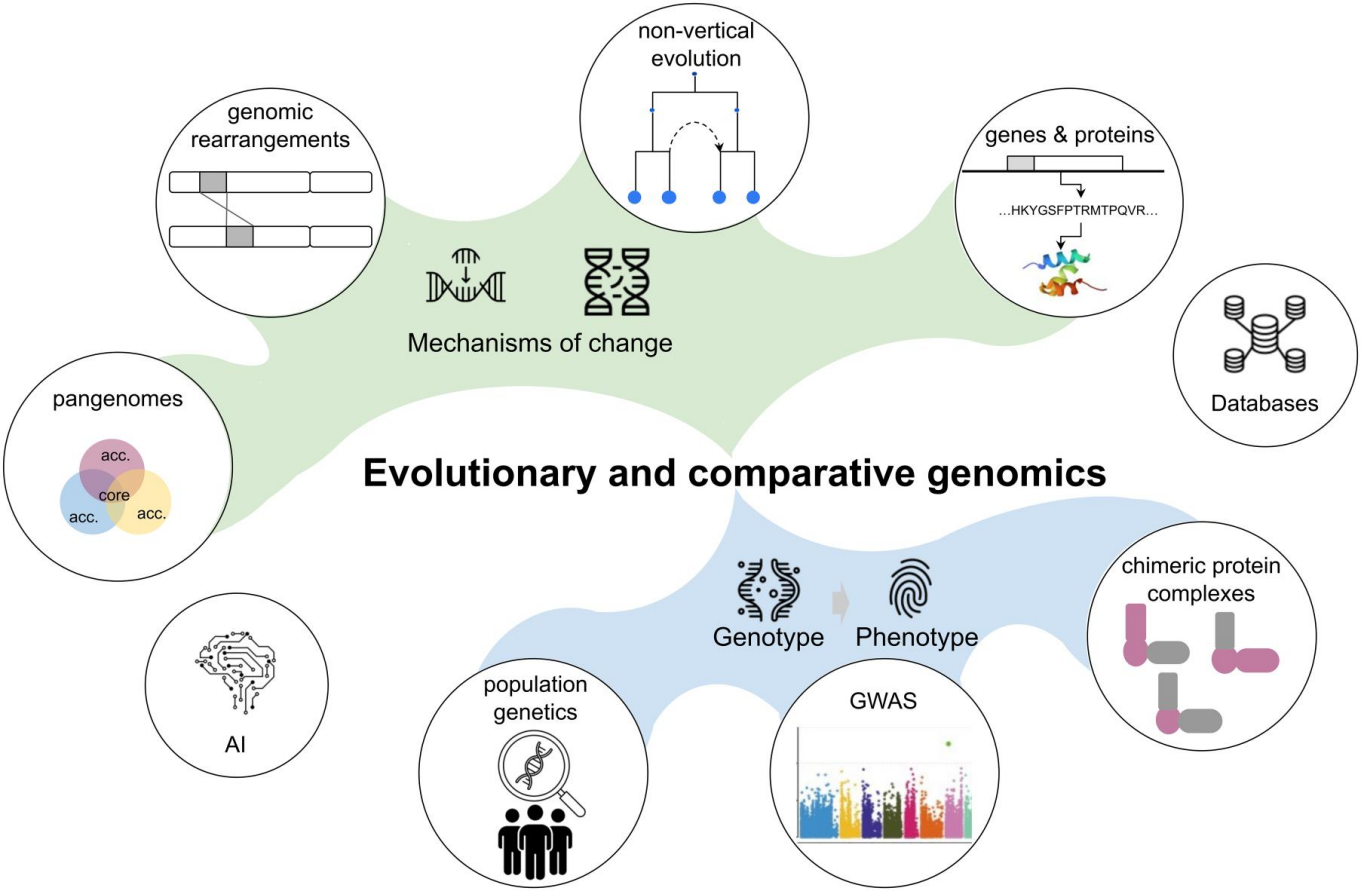
Summary of topics. The meeting on evolutionary and comparative genomics revolved around two broad questions: firstly, how do genomes change over time (green shape), which was addressed through studies of pangenomes, genomic rearrangements, non-vertical evolutionary mechanisms and changes in gene and protein sequences. Secondly, how do genotypes translate to phenotypes (blue shape), a topic explored through population genetics, GWAS, and targeted studies of specific molecular phenotypes, such as chimeric protein complexes. Icons by Irfan ms, NIXX Design, Hendrik Hermawan, Vectorstall, Hadi Bagaji, Edy Susanto, Arif Hariyanto and chonri510 from Noun Project (https://thenounproject.com/, CC BY 4.0). GWAS plot adapted from Figure 3 of Yin et al (2021), https://doi.org/10.1016/j.gpb.2020.10.007. Protein structure taken from PDB (see DOI:10.2210/pdb5UOI/pdb).

We collected the overarching topics discussed (Fig. 1) and in the following sections we summarize and comment on the key takeaways of presentations from both the main and satellite event.

## 2 Core insights

### 2.1 Abundant, open data as a crucial resource

The explosion of biological data presents significant opportunities for novel computational analyses that can address a plethora of open questions in the life sciences. Now more than ever, it is important to ensure that data are accessible and organized in a way that facilitates and bolsters downstream analyses. A lot of effort has already been invested to this end, as evidenced repeatedly by work presented in this meeting, which in many cases introduced, contributed to, or utilized well-structured databases.

A prime example was the databases resulting from a consistent effort to catalogue the microbial protein universe, through mega-scale analyses of all available bacterial genomes and metagenomes, as presented by Nikos Kyrpides. This effort has made available a vast resource of microbial protein families, the vast majority of which are unknown and of which a significant percentage are restricted to specific taxonomic groups and environments (Pavlopoulos *et al.* 2023). A similar kind of approach had previously generated the Unified Human Gastrointestinal Catalogue, an exhaustive list of all the genes and protein families that can be found in prokaryotes living in the human gut (Almeida *et al.* 2021).

This was the case of Rosa Fernández who presented a homogeneous genomic, transcriptomic and functional database comprising representatives of the entire animal diversity (MATEDB) (Martínez-Redondo *et al.* 2024). Another example was the meta-catalogue of consistently translated human small ORFs (Mudge *et al.* 2022), assembled by a consortium including Mar Alba, who introduced this resource in her lecture.

Most notably, the presentation of Antonis Rokas focused on the Y1000+ Project, which recently released genomic, phenotypic, and environmental data from nearly all >1000 known yeast species in subphylum Saccharomycotina (Opulente *et al.* 2024). Using this treasure trove of data, his lab, together with their collaborators are linking genotype to phenotype (and *vice versa*) using a wide variety of approaches (e.g. phylogenetic profiling, phylogenetic regression, and random forest models) for a wide diversity of traits, including metabolic niche breadth (Opulente *et al.* 2024), galactose utilization (Harrison *et al.* 2024), and cactophily (Gonçalves *et al.* 2024).

Other lectures also highlighted the abundance of high quality data of various types, such as long-read based genome assemblies and transcriptomes (Schmitz *et al.* 2018). Iliana Bista presented the important work of large consortia generating reference genomes from across the tree of life. Especially the work of the Vertebrate Genomes Project (VGP) (Rhie *et al.* 2021) and the Darwin Tree of Life (DToL) project whose data are fully accessible  (Darwin Tree of Life 2025). These initiatives along with others are part of the Earth Biogenome Project (EBP) which is an umbrella initiative and have been characterized as “a moonshot for biology” (Lewin *et al.* 2018). Furthermore, she discussed standardization of annotations to facilitate inter-study comparisons, for example of transposable elements (Peona *et al.* 2024).

The impact of existing resources is already evident, as they have enabled groundbreaking discoveries and new avenues of investigation. As more research continues to build upon these datasets, their importance will only grow. However, while generating sequencing data has become easier, ensuring its long-term usability remains a challenge. Efforts to curate, standardize, and maintain databases are crucial for maximizing their potential, yet they often face funding limitations. Moving forward, sustained investment in these efforts will be essential to take full advantage of the power of open biological data.

### 2.2 Artificial intelligence

As in numerous other scientific disciplines, (and indeed, many other aspects of human activity) the impact of deep learning on biology has been substantial. From making millions of structures available for all types of comparative analyses (Jumper *et al.* 2021), to enabling remote homology detection (Hamamsy *et al.* 2024), to understanding complex phenotypes (Opulente *et al.* 2024), or substantially accelerating computationally challenging phylogenomic problems.

Alexandros (Alexis) Stamatakis presented his vision for improving phylogenetic analyses by incorporating uncertainty assessment at every stage and propagating the respective uncertainty to subsequent phylogenetic inference pipeline stages, that is, from orthology assessment, over multiple sequence alignment, to tree inference. Since methods to assess uncertainty are not yet readily available for every pipeline stage and the naive propagation of uncertainty induces an exponential increase of downstream computations, he presented recent developments for uncertainty assessment in the area of phylogenetic inference. To this end, he introduced Pythia (Haag *et al.* 2022), a tool to predict the difficulty (i.e. the signal strength) of a likelihood-based phylogenetic inference given the input MSA, prior to executing the actual tree inference. Pythia thereby allows to devise an appropriate analysis strategy and adjust expectations about support values prior to executing the actual tree inference. He further presented adaptive RAxML-NG, a novel tree search heuristic that automatically adapts the thoroughness of the search and hence the computational effort being invested as a function of the Pythia difficulty score of the alignment (Togkousidis *et al.* 2023). Finally, Stamatakis also presented the Educated Bootstrap Guesser, a novel, fast, and accurate tool for predicting bootstrap support values via machine learning (Wiegert *et al.* 2024) and Pandora, a tool for assessing the inherent uncertainty of dimensionality reduction methods such as PCA or MDS as used in population genetic studies (Haag *et al.* 2025). Overall, Alexis Stamatakis’ seminar gave an overview of methods that can assess uncertainty in evolutionary genomics analyses, as well as machine learning approaches that take into account this uncertainty to perform standard phylogenetic inference tasks in a faster and data-adaptive manner.

The revolutionary potential of deep learning was also exemplified by Rosa Fernández’s presentation of FANTASIA, a pipeline that integrates protein language models for large-scale functional annotation of proteins that are beyond the reach of traditional sequence similarity based approaches (Barrios-Núñez *et al.* 2024, Martínez-Redondo *et al.* 2025).

AI has undeniably expanded the possibilities of computational biology, enabling analyses that were previously infeasible due to complexity or computational constraints. However, as the presentations in this meeting demonstrated, the true driving force behind progress is not AI itself, but the creative scientists who harness its potential in innovative and unexpected ways. The most significant advancements will come not just from more powerful algorithms, but from researchers who can push the boundaries of how these tools are applied, turning abstract models into meaningful biological insights. In this sense, AI is not a replacement for human ingenuity but an amplifier of it—its greatest impact will be realized through the vision and curiosity of those who wield it.

### 2.3 Mechanisms of evolutionary change

As AI and data-driven approaches reshape bioinformatics, the core of evolutionary research remains the study of the mechanisms driving genetic and phenotypic change. This section explores key innovations in evolutionary genomics, from molecular processes of new gene origination to large-scale genomic rearrangements and regulatory dynamics. By combining computational advances with emerging sequencing technologies, researchers are uncovering the forces that shape biodiversity and adaptation.

#### 2.3.1 Evolution of new genes and protein functions

On the molecular level, Mar Albà presented her project on microproteins, a potential source of novel proteins. She argued that there is an overlooked abundance of these small Open Reading Frames (sORFs) in eukaryotes, which are often missed by standard annotation methods due to their small size and possible misidentification as untranslated regions. Additionally, Albà highlighted upstream Open Reading Frames (uORFs) as a source of new microproteins with a unique amino acid bias (Moro *et al.* 2021). Her research integrates transcriptomics and translatomics to study small genes that originate de novo from non-coding regions in species such as human or yeast (Blevins *et al.* 2021), as well as more the various regulatory roles of uORFs (Ruiz-Orera and Albà 2019).

Following up, Erich Bornberg-Bauer discussed theories on protein function evolution, contrasting the traditional view of gradual adaptation following gene duplication with the processes of modularity and de novo emergence from previously non-coding regions. He proposed that short genes are tolerated by cells due to either their rapid degradation and because older proteins have an evolutionary acquired tendency to tolerate new ones in general and some adapt to interacting with them. This bides them time and allows new proteins to persist until they have been assigned a functional role, which might explain why many *de novo* proteins show next to no signs of adaptation, at least on shorter time scales. Furthermore, using the analogy of a shallow “golf course,” he emphasized that the folding, and therefore also the functional landscape of proteins allows for many viable states which can be interconverted without detrimental intermediate states. He emphasized the importance of protein domain synteny for structure evolution. He underlined the adaptive efficiency of modular protein evolution, likening them to toy building blocks, which can be recombined to create new functions (Bornberg-Bauer and Mar Albà 2013). At a more elementary level, these may have emerged early in life’s history and are now reused combinatorially.

#### 2.3.2 Non-vertical gene evolution

Larger-scale rearrangements of existing genetic elements can also take place, increasing the complexity of comparative analyses. Chris Jiggins presented recent research on the prevalence of hybridization and introgression between species, even where hybrids are rare, focusing on South American butterfly populations that exhibit distinct ecological traits due to multilocus introgression (Wallbank *et al.* 2016). He discussed the challenges of using genome scans to identify ‘speciation genes’ and the various processes that influence patterns of Fst, which can potentially be overcome using modelling approaches such as gIMble (Laetsch *et al.* 2023). Genomics has been a powerful approach to study barriers to gene flow and hybrid population dynamics, offering insights into speciation mechanisms, and has changed our view of the “tree of life” as being more of a complex network.

In adherence with evolutionary trajectories that are incompatible with binary trees, a notable highlight was Nick Irwin’s work on horizontal gene transfer (HGT) in eukaryotes, (which he presented at the satellite symposium) where he showed how bacterial genes enabled innovations like phytohormone production and secondary metabolism in plants and algae. He also described repeated ancient transfers of bacterial genes, which have evolved distinct functions in plants, animals, and green algae. His findings highlighted the role of HGT in driving complexity across lineages.

#### 2.3.3 Genomic architecture and dynamics

While genetic rearrangements and small-scale mutations provide the raw material for evolutionary change, the regulation of gene expression and the dynamic nature of genomes play an equally crucial role in shaping phenotypic outcomes and driving adaptation. Pointedly, transposable elements (TEs) are important actors of evolution providing a source of new regulatory structures or disrupting existing ones. Iliana Bista gave an overview of the contribution of transposable elements in genome evolution and the importance of accurate transposon annotation across diverse taxa, advocating for standardized metrics to facilitate inter-study comparisons, taking into consideration the complex variation of TEs. The study of transposons across the tree of life was limited in the past due to the lack of appropriate genomic data. Advances in long read sequencing, and the contributions of large genome sequencing initiatives such as the Vertebrate Genomes Project (VGP) (Rhie *et al.* 2021), and Darwin Tree of Life (DToL) ( Darwin Tree of Life 2025)  are revolutionizing the field. Bista presented her work as part of the VGP and the evolution of Antarctic notothenioid fish, a radiation adapted to the freezing waters of the Southern Ocean (Bista *et al.* 2023). Using long reads enabled deciphering of evolutionary history of complex genomic regions such as antifreeze genes and hemoglobins, which was linked to transposon activity also causing a large genome expansion across the radiation (Bista *et al.* 2023, Bista and Collins 2024, Desvignes *et al.* 2023).

Shifting into a different class, Camille Berthelot explored how gene regulation drives the emergence of new traits in mammals, using multiomics approaches to investigate enhancer evolution and quantitative trait analyses (Parey *et al.* 2023). She emphasized the rapid evolution of enhancers in mammals and their pivotal role in shaping biodiversity. She presented how quantitative approaches to model trait evolution can be adapted to functional genomic data in order to identify putative episodes of positive selection on gene expression and enhancer activity. More importantly, her work showcased how the enhancer-orchestrated, complex regulatory behavior can be conserved in a way that is disconnected from sequence conservation, shedding new light on genotype-to-phenotype evolution.

Switching to another model system, Rosa Fernández used comparative genomics and genome architecture approaches to study the massive disruption of macrosynteny between marine and non-marine annelids (clitellates). She discussed how the loss of macrosynteny was coupled to a loss in orthologous groups related to genome stability and homeostasis, and how this potentially triggered a series of rare genome-wide events such as several rounds of whole genome duplication or extensive rearrangements between clitellate lineages (Vargas-Chávez *et al.* 2024). Her work emphasized the 3D genomic changes associated with large-scale genome innovation, a phenomenon she referred to as “chromoanagenesis” (Pellestor *et al.* 2022), as well as their potential role in the colonization of terrestrial environments in clitellates, formulating hypothesis about the genomic basis of adaptation in terrestrial animals.

Gilles Fischer presented work on the structural diversity of Lachancea yeast genomes, emphasizing the use of the CHROnicle suite of tools developed in his group that enable the comparison of genome architecture and rearrangements between species, including the quantification of conserved synteny (SynCHRO) (Drillon *et al.* 2014) and ancestral genome reconstruction (AnCHRO) (Vakirlis *et al.* 2016). Fischer discussed how the integration of analysis on different levels of molecular evolution in the Lachancea genus of yeasts revealed that the rate of point mutations, rearrangements and gene family expansions appear to reach fixation at a coordinated pace (Vakirlis *et al.* 2016).

#### 2.3.4 Intraspecific genomic diversity

Building on the understanding of how genomes are dynamically shaped, we now turn to the diverse outcomes of these processes: the vast array of genomic diversity that drives evolutionary innovation and adaptation and how it is perceived. The results of these changes, such as whole genome duplications and gene content variations, can now be detected with bioinformatics methods. Bacteria have particular dynamic genomes, due to frequent horizontal transfer of genes between organisms in this group. This results in large pangenomes, i.e. all groups of homologous genes, within a bacterial species. The talk of Anne Kupczok emphasized the significance of synteny in improving orthology inference, which in turn enhances downstream pangenome analyses (Tonkin-Hill *et al.* 2020). She presented the new gene coincidence analysis tool, Goldfinder, which takes phylogenetic relationships into account to identify biologically meaningful associations among accessory genes (Gavriilidou *et al.* 2024). On the same topic, Grigoris Amoutzias presented a pangenome-based method/pipeline named pyPGCF that performs phylogenomic studies of hundreds to thousands of prokaryotic genomes/proteomes (maximum likelihood trees of the analysed genomes/proteomes based on the concatenated alignments of their core gene/protein sets) and identifies lineage-specific core genes (termed fingerprints) that distinguish prokaryotic evolutionary lineages and provide clues about which genes, pathways and functions may be involved in such adaptations (Nikolaidis *et al.* 2024). He further demonstrated how this methodology revealed lineage-specific adaptations related to the lifestyle and pathogenicity in key bacterial genera and their species, such as Pseudomonas (Nikolaidis *et al.* 2020), Bacillus (Nikolaidis *et al.* 2022), and Streptomyces (Nikolaidis *et al.* 2023).

Intraspecific genomic diversity can manifest in unexpected ways even in short timescales: as Gilles Fischer presented, in their experiments with budding yeast they found that during the normal development of a colony, a small subpopulation of cells enter a transient mutator state (ie acquire an increased mutation rate) that allows them to accumulate multiple mutations, including genomic rearrangements, during a short period of time before the mutator state is reverted back to normal, as it was not genetically encoded but only phenotypically induced ( Agier *et al.* 2025).

### 2.4 Genetic basis of phenotypic adaptations

Understanding the mechanisms of evolutionary change provides a foundation for exploring how these processes manifest in the traits and adaptations that define life’s diversity. At the core of these adaptations lies the genetic basis that drives the emergence, refinement, and persistence of phenotypic traits. This section delves into how genomic changes translate into functional outcomes, enabling species to adapt to their environments and evolve new capabilities.

#### 2.4.1 Emergence of adaptive innovations

By uncovering the genetic foundations of these adaptations, researchers can gain insights into how traits evolve, how organisms respond to environmental challenges, and how evolutionary processes shape biodiversity over time. Daniela Delneri created complex interspecific yeast hybrids able to produce meiotic progenies and investigated the effect of trait recombination on phenotype (Naseeb *et al.* 2021, Visinoni *et al.* 2024). She also highlighted the influence of mitochondria on Quantitative Trait Loci (QTLs), noting that hybrids with the same nuclear genome but different mitochondria rarely share QTLs (Naseeb *et al.* 2021). Her team explored the natural establishment of chimeric protein complexes in both lab and natural hybrids and explored their effect on adaptation and pattern of gene loss (Piatkowska *et al.* 2013, Timouma *et al.* 2021). Additionally, many of the studies described in previous sections shared insights on adaptive innovations, be it evolution of regulatory (Parey *et al.* 2023) or transposable elements (Bista and Collins 2024), or emergence of traits via horizontally acquired or de novo genetic elements (Vakirlis and Kupczok 2024).

#### 2.4.2 Complexity of the genotype-phenotype map

The study of adaptive innovations lays the groundwork for understanding how evolution drives increasingly complex traits and patterns. With the growing availability of large-scale genomic datasets and advanced analytical tools, researchers can now tackle the intricacies of complex traits and uncover striking examples of convergent evolution, offering deeper insights into the shared and unique pathways that shape life’s diversity. Joseph Schacherer focused on the genetic basis of complex traits, leveraging GWAS and coexpression networks to study transcriptome-proteome variation (Teyssonnière *et al.* 2024). He introduced the concept of expression and protein quantitative trait loci (eQTL and pQTL) to connect genetic variation to phenotypic outcomes. Schacherer also argued that ancestral genomes were simpler than current models suggest, possibly due to the decreased strength of modern inference algorithms to identify phylogenetically deep connections.

While the study of complex traits reveals the intricate genetic and environmental interactions underlying adaptation, the examination of convergently evolving traits offers a unique perspective on how similar solutions can arise independently across diverse evolutionary lineages, shedding light on shared selective pressures and molecular pathways. Antonis Rokas emphasized the importance of convergent evolution in understanding how genomic variation transforms into phenotypic variation over macroevolution and stressed the necessity of well-defined datasets for studying convergent evolution and its genetic architecture. Apart from his work on yeast (described earlier), there were two more projects presented, which focused on convergently evolved complex traits. Athina Gavriilidou demonstrated how phylogenetic profiling can be applied to study the evolution of venom systems, exploring indications of co-evolution between genes related to toxins and resistance mechanisms. Her work highlighted potential unknown interacting proteins, including venom components and putative self-immunity mechanisms. Building on the theme of molecular evolution in animals, Alexandros Pittis explored the origins of animal neural systems, tracing the origins of proto-neuronal machinery and demonstrating how neurotransmitter pathways arose from ancestral promiscuous mechanisms. His work on olfactory receptors further revealed the molecular evolution of sensory systems, showcasing how comparative genomics can illuminate complex biological traits.

#### 2.4.3 Evolution in context

The evolution of complex traits does not occur in isolation but is shaped by a dynamic interplay between genetic change and external influences. It is therefore equally important to consider how environmental pressures and ecological interactions contribute to evolutionary trajectories. One example from the microbial world was highlighted by Nikos Kyrpides with his work on microbiomes and the so-called “functional dark matter” (Pavlopoulos *et al.* 2023). This study revealed an enormous and previously hidden reservoir of novel functional diversity in microbial communities. In a separate line of work currently unfolding, his team has identified that a large number of uncultivated organisms lack core metabolic pathways, such as those for amino acid or cofactor biosynthesis, suggesting that these microbes rely on metabolic exchanges and syntrophic interactions within their communities (Olo Ndela 2025). Together, these findings point to the importance of metabolic complementarities as a key organizing principle in microbial ecosystems. From a population genomics point of view, Tereza Manousaki showed how investigations of population structure and loci-environment associations in marine species like sardines and lionfish (Blakeslee *et al.* 2020) contribute to our understanding of seascape genomics.

Beyond contemporary ecological and environmental influences, historical processes shifting our perspective to human and pathogen palaeogenomics. Eirini Skourtanioti provided an overview of how ancient DNA analysis has advanced our understanding of human mobility, admixture, and genetic adaptation, particularly during major cultural and technological transitions of the Holocene (∼10 000 yBP). Skourtanioti focused on archaeogenetic research in the Aegean, from the first farmers (ca. 6000 BC) to the complex Bronze Age civilizations of the Cyclades, Crete (“Minoan”), and Greek mainland (“Mycenaean”) (ca. 3100–1200 BC). These cultures thrived on exchange networks, but the role of human mobility in sociocultural change remained unclear. Analysis of over 100 genomes revealed distinct gene flows including from populations to the north linked to Pontic steppe pastoralists (Lazaridis *et al.* 2017, 2022, Clemente *et al.* 2021, Skourtanioti *et al.* 2023). Additionally, the “Pontic steppe” genetic influence spread from the mainland to Crete, shedding light on the political dynamics following the collapse of Minoan culture (Skourtanioti *et al.* 2023).

#### 2.4.4 Perspectives on health and disease

The study of evolution not only deepens our understanding of biodiversity and adaptation, but also has direct implications for human health, disease ecology, and pathogen evolution. The discovery of novel genes, including small open reading frames (sORFs) and upstream open reading frames (uORFs), highlights how genomes continuously generate new functional elements, some of which may play an important role in disease (Moro *et al.* 2021). Pathogens evolve through similar processes, with fungi independently acquiring virulence traits and RNA viruses demonstrating remarkable plasticity (Mead *et al.* 2019, van Valkengoed *et al.* 2025). These pathogens are detectable through environmental sequencing or can be found incidentally in public sequencing data sets, highlighting again the importance of open biological data. Ancient DNA research extends this perspective to human disease history, showing how malaria and other pathogens shaped populations over millennia, sometimes preceding historical records (Michel *et al.* 2024). These examples demonstrate how comparative genomics does not only uncover the genetic mechanisms behind adaptation but also offer valuable tools for understanding and addressing current and future challenges in human/animal/plant health, evolution, and society.

## 3 Outlook

The EMBO Evolutionary and Comparative Genomics meeting was more than just a gathering of researchers—it was a reflection of the current momentum in the field. From data accessibility and AI-driven analyses to the molecular and genomic mechanisms driving evolutionary change, the discussions highlighted not only where we stand but also where we are headed. One of the most valuable outcomes was the realization that, despite working on diverse systems—from viruses and bacteria to vertebrates, from methods development to applied genomics—we share common challenges and conceptual frameworks. The exchange of ideas across disciplines and study systems proved to be an engine for innovation, revealing new connections across the tree of life.

As the field advances, the need for integration across scales—from molecular evolution to ecosystem-level processes—becomes even more pressing. The increasing availability of genomic data and the computational power to analyze it are transforming our ability to ask and answer fundamental evolutionary questions. However, these advances also require thoughtful discussions about standardization, accessibility, and meaningful interpretation of complex data. The meeting underscored the importance of open dialogue and collaborative efforts in tackling these challenges, fostering an environment where diverse perspectives can shape the future of evolutionary genomics.

Future iterations of this meeting should build on what made the first edition successful: bringing together researchers from different backgrounds, encouraging dynamic discussions, and embracing the breadth of topics within evolutionary and comparative genomics. While maintaining inclusivity, future editions could also explore thematic focuses—whether on emerging methodologies, the role of genomics in human and environmental health, or fundamental evolutionary questions like the evolution of novelty and genome architecture. Regardless of the specific themes, the key takeaway remains: evolutionary genomics is not just about accumulating data but about using it to uncover the principles that shape life’s diversity. This meeting demonstrated the power of combining diverse perspectives, and sharing these insights more broadly ensures that the conversations sparked here will continue to inspire new discoveries.

Feedback on the scientific content and format of the meeting was strongly positive, with almost everyone appreciating how substantial, direct and frequent the dialogue was among participants. Attendees particularly benefited from the small size and the conviviality of the event, where asking questions during the lectures was highly encouraged (earlier-stage attendees were given priority) and approaching more senior researchers was seamless since everyone was present and following the same schedule most of the time. One strength of this meeting was that it brought together researchers working on substantially different organisms and gave them the opportunity to draw inspiration from one another’s problems and solutions.

## 4 Practical overview of the event

The 3-day event was made possible by a grant from the European Molecular Biology Organization (EMBO) and was organized by Dr. Nikolaos Vakirlis (main organizer; BSRC “Alexander Fleming”), and co-organizers Dr. Christoforos Nikolaou (BSRC “Alexander Fleming”), Dr. Tereza Manousaki (HCMR), Dr. Alexandros Pittis (IMBB-FORTH) and Prof. Grigoris Amoutzias (University of Thessaly). The course attracted 48 participants, which together with 18 invited speakers and organizers gave a total of 66 attendees. The program of the meeting consisted mostly of sessions of 50-minute lectures which combined accessible background and introductory concepts with the latest cutting-edge research. Speakers and organizers actively encouraged questions during the lectures, and participants duly responded. This created a positive, relaxed atmosphere which in turn led to increased interactions. Key to that was the size of the room which was ideal for the number of people and allowed direct questions without a microphone. The program consisted mostly of sessions of fifty-minute lectures which combined accessible background and introductory concepts with the latest cutting-edge research. Each session ended with a round-table discussion between the speakers and the audience. In every single one there were more questions than time to answer them. Forty-three works were presented as posters, including research on organisms ranging from well-established models like budding yeast and fruitfly, to aphids, bats, crop plants, ciliates, fish, poison frogs, gulls, phytoplankton, and many more. It is no exaggeration to say that, as a whole, the poster presentations constituted one of the highlights of the meeting. The very high overall quality of the work presented yielded voting for poster prizes a tough task, but in the end three prizes were given out: to Gauthier Brach (University of Strasbourg, France) for his work entitled “Exploration of transcript diversity in a yeast natural population using direct RNA sequencing,” to Anastasia Christinaki (University of Athens, Greece) for her work entitled “Comparative genomic analysis of fungicolous secondary metabolism and pathogenicity: insights from four newly sequenced genomes” and to Yu-Chen Yue (Academia Sinica, Taiwan) for his work entitled “Disentangling the biogeography and ecological roles of Saccharomycotina yeasts.”

Apart from lectures and poster sessions, we held a speed-networking session during breakfast of the last day with active participation from most of the meeting’s attendants. The provided social event was a dinner featuring local cuisine from the region of Argolis, which everyone seemed to enjoy. One morning was free and used by attendees to either explore the city of Nafplion or visit the nearby archeological site of Mycenae. The satellite meeting reached its maximum capacity of 80 registrants, most of which came from the wider Athens region.

## Data Availability

No new data were generated or analysed in support of this research.
